# Dietary polyphenols as modulators of cell signaling and inflammation in colorectal carcinogenesis

**DOI:** 10.3389/fnut.2025.1691590

**Published:** 2025-11-14

**Authors:** Bee Ling Tan, Fatimah Zulkifli, Mohd Esa Norhaizan

**Affiliations:** 1 Department of Diagnostic and Allied Health Science, Faculty of Health and Life Sciences, Management and Science University, Shah Alam, Selangor, Malaysia; 2 Department of Nutrition, Faculty of Medicine and Health Sciences, Universiti Putra Malaysia, Serdang, Selangor, Malaysia

**Keywords:** antioxidant, colorectal cancer, flavonoid, inflammation, oxidative stress, polyphenol

## Abstract

Colorectal cancer is the second leading cause of cancer mortality worldwide. Although current chemopreventive drugs can suppress the proliferation of cancer cells, their use is often limited by adverse side effects, including toxicity, development of resistance, and lack of selectivity. The main side effects associated with continuous infusion of 5-fluorouracil (5-FU) are mucositis, vomiting, nausea, and diarrhea. Dietary factors play a critical role in colorectal cancer management and have gained attention as alternative strategies for cancer prevention. Evidence from preclinical and human studies suggests that polyphenols exert anti-colon cancer activity. However, the mechanisms underlying these effects remain poorly understood. This review highlights the potential of polyphenol-rich foods in the prevention of colorectal cancer, particularly regarding molecular aspects that may provide a plausible means for the prevention of colorectal cancer. Overall, elucidating the role of polyphenols in redox regulation of inflammation may offer useful strategies for intervention and contribute to colorectal cancer prevention.

## 1 Introduction

Colon cancer represents the second biggest cause of cancer-related deaths globally (1). In 2020, there were almost 1.9 million new instances of colorectal cancer worldwide, with 930,000 deaths. Geographical differences were observed in both incidence and mortality rates. The majority of cases occurred in Europe, Australia, and New Zealand, with the highest fatality rates in Eastern Europe. According to the WHO (1), by 2040, there would be 3.2 million new cases of colorectal cancer (63% increase) and 1.6 million deaths (73% increase) from the current rate. In general, colorectal cancer originates in the colon or rectum. It is among the most frequent malignancies worldwide (1). A benign polyp (adenoma) can progress to dysplasia and cancer through genetic and epigenetic alterations over time (2). This condition is sporadic and linked to risk variables including age, ethnicity, food, and inflammatory bowel disease. Lynch syndrome and genetic polyposis syndromes are common causes of inherited colon cancer (3).

Inflammation constitutes an innate protective reaction triggered by harmful conditions such as microbial invasion, cellular stress, or tissue damage. Chronic inflammation is closely linked to the development and progression of various chronic diseases, including cancer (4). Overproduction of pro-inflammatory cytokines (Interleukin 6 [IL-6] and tumor necrosis factor [TNF-α]) and enzymes (COX-2 and iNOS) has been implicated in the development of malignancies in the colon (5). Given the pivotal role of inflammation in colorectal cancer, therapeutic strategies have focused on targeting pro-inflammatory mediators. Despite extensive efforts to enhance current therapeutic strategies, conventional therapy is not likely to be effective due to the undesirable side effect profiles of these agents (6). Accordingly, there is a critical need to identify and develop agents that are both safe and efficacious in preventing and alleviating colorectal cancer. Chemoprevention in cancer involves using natural, synthetic, or biological compounds to interfere with the initiation of carcinogenesis and to impede the advancement of premalignant cells toward malignant disease (7).

Phytochemicals derived from terrestrial plants have been documented to exert protective effects against the development of colorectal cancer (8). Dietary polyphenols, such as catechins, anthocyanins, quercetin, and resveratrol, have been demonstrated to alleviate colonic inflammation *in vivo* (9). Therefore, polyphenols are regarded as promising agents for attenuating colorectal cancer. Nonetheless, the modes of action through which polyphenols exert their protective effects remain incompletely elucidated. In this review, special attention is given to the molecular mechanisms by which dietary polyphenols contribute to the prevention of colorectal cancer.

## 2 Etiological factors

### 2.1 Genetic and inherited risk factors

Hereditary colorectal cancer accounts for approximately 5%−10% of all colorectal cancer cases (10). Genetic identification is essential for managing these diseases, which have unique symptoms and routes of inheritance. The majority of colorectal cancer cases are sporadic, although the most prevalent inherited variants are familial adenomatous polyposis (FAP) and Lynch syndrome (11). Hereditary colorectal cancer accounts for approximately 5%–10% of all colorectal cancer cases, primarily due to pathogenic mutations in inherited syndromes such as familial adenomatous polyposis and Lynch syndrome, which exhibit higher penetrance for certain mutations. Goosenberg et al. (12) suggested that genetic testing can assist in identifying individuals at risk and in developing personalized prevention and treatment measures.

According to Karstensen et al. (13), FAP is the most common adenomatous polyposis syndrome and one of the rarest autosomal dominant transmission illnesses. This condition is characterized by hundreds to thousands of adenomatous polyps in the gastrointestinal mucosa, as well as several extraintestinal symptoms. Individuals with FAP are most likely to acquire colorectal cancer over their lifespan. FAP is defined as either classic or attenuated. Attenuated FAP (AFAP) is a milder presentation that arises later in life, with fewer polyps (typically 0 to 100) and a lower lifetime colorectal cancer risk (60%−80%), with a preference for the right hemicolon (11). Early diagnosis and vigorous treatment of FAP are crucial due to its high cancer risk. Indeed, over 90% of FAP patients were positive for adenomatous polyposis coli (APC) mutations (14).

Adenomatous polyposis syndromes account for approximately 1% of all hereditary colorectal cancer syndromes and are linked to mutations in the APC gene (dominant inheritance) and the MUTYH gene (recessive inheritance). Lynch syndrome, which accounts for 2.3% of all colorectal cancer cases, is caused by germline or epistatic mutations in mismatch repair (MMR) genes such as MLH1, MSH2, MSH6, and PMS2 (12). Hamartomatous polyposis syndromes are rare (< 0.1%) and linked to mutations in the STK11, PTEN, BMPR1A, and SMAD4 genes (11).

### 2.2 Environmental and lifestyle factors

Colorectal cancer is suggested to be caused by a combination of genetic, environmental, and lifestyle factors. Epidemiological studies have shown that nutrition, physical activity, smoking, alcohol consumption, and obesity are risk factors for colorectal cancer (15). Modifiable factors offer opportunities for effective preventative interventions, especially as colorectal cancer incidence rises in certain Asian regions, such as Malaysia.

Diet has a key role in colorectal cancer formation. Research repeatedly links excessive consumption of red meat and processed meat, poor dietary fiber intake, alcohol use, and a Western diet to an increased risk of colorectal cancer (16). Nonetheless, dietary components, including whole grains, fruits, vegetables, dairy, and calcium supplementation, offer protection. Dietary patterns, rather than individual foods or nutrients, have a significant impact on the risk of colorectal cancer. Nutritional epidemiology has shifted from studying specific foods to examining dietary patterns as a whole to better understand the impact on health outcomes. This is particularly important in multi-ethnic countries such as Malaysia, where different food traditions coexist. Factor analysis has been used to identify dietary patterns associated with colorectal cancer in various groups. Despite the growing concern, few studies have investigated how dietary intake contributes to the development of colorectal cancer risk in Malaysia (17, 18). Studies indicate a transition from traditional high-fiber plant-based diets to Westernized diets high in processed foods, sweets, and saturated fats, which is linked to rising colorectal cancer incidence (19).

### 2.3 Gut microbiota and inflammation

Chronic inflammation plays a pivotal role in the pathogenesis of various diseases, such as cancer. Cancer associated with chronic inflammatory bowel diseases (IBD) and colitis-associated colorectal cancer (CA-CRC) risk was found to be higher in patients with chronic IBD such as ulcerative colitis (UC) and Crohn's disease (CD) (20). This inflammation may stimulate cancerogenesis through the release of bioactive molecules such as cytokines, growth factors, and chemokines that can carry proliferation and cell survival signals together and promote neo-angiogenesis. As a result, they disrupt metabolic processes, trigger inflammation, and alter cancer-related genes. Dysplasia of intestinal epithelial cells, including increased cell proliferation and cell death, can lead to disease development (21).

The gut flora plays a crucial role in the development of colorectal cancer. Previous studies indicate a substantial link between colorectal cancer and intestinal dysbiosis (22). The gut microbiota is disrupted by dysbiosis, reducing beneficial microbes and increasing dangerous bacteria. Dysbiosis in gut microbiota can produce harmful metabolites, such as carcinogenic and pro-inflammatory chemicals, thereby contributing to colorectal cancer development (23). Subsequently, the mucosal barrier can be damaged by dysbiosis, enabling toxicants, and the toxins produced by the bacteria to get through, leading to an inflammatory response that promotes cancer growth and spread (24).

Dysbiosis in colorectal cancer may also affect the cancer's microenvironment (25). Research indicates that some bacteria in the gut microbiome play a role in the development of colorectal cancer. Alistipes, a gut-resident bacterium, has been linked to colorectal cancer progression. The bacteria produce nitrosamines, which can be harmful to the colon mucosa (26, 27). The development of colorectal cancer is contributed to through these metabolites (28). Intriguingly, modifying the gut microbiome can help prevent and treat colon cancer (29). Modifying the gut microbiota might reduce hazardous microbes and enhance helpful bacteria, potentially preventing colorectal cancer (30). Therefore, consuming probiotics and prebiotics can help prevent and treat colorectal cancer (26).

Fiorentini et al. (31) linked gut microbiota dysbiosis and inflammation to the development and progression of colorectal cancer. Dysbiosis alters host inflammation-related genes in the gut (32). Modulating the gut microbiome may alter inflammatory responses. Ma et al. (26) proposed using Mendelian randomization (MR) to evaluate the causal link between risk factors and diseases. This method uses single-nucleotide polymorphisms (SNPs) associated with risk factors as independent variables. Genetic variation within the zygote can help resolve confusion and improve selectivity in MR studies (33).

## 3 Molecular pathogenesis of colorectal cancer

### 3.1 Chromosomal instability (CIN) pathway

Approximately 80% of colorectal cancer originates through the chromosomal instability (CIN) route (34). Tumors with somatic copy number alterations (SCNA) and structural chromosomal alterations (losses, amplifications, aneuploidy, and translocations) are associated with changes in genes such as APC, KRAS, SMAD4, or TP53 but are not hypermutated (35). CIN pathway colorectal cancers that arise from ordinary adenomas typically follow the CIN pathway. Clinically relevant signaling for CIN includes the Wnt and MAPK pathways that contain additional molecules such as beta-catenin, which accumulates in the nucleus and initiates transcription. It may take more than 10 years for cancer to develop as a result of CIN (36).

The CIN pathway is present in 65%−70% of recurrent colorectal cancer, characterized by chromosomal alterations such as SCNA caused by aneuploidy, deletions, insertions, and amplifications. Nguyen et al. (34) found that non-hypermutated tumors from this route exhibit fewer base pair mutations in coding regions. CIN can be caused by chromosomal instability, such as sister chromatid segregation (37), decreased cell senescence due to telomere shortening and genomic rearrangement, and malfunctioning DNA damage response (DDR) (37).

These karyotypic changes are often concomitant with mutations of the tumor suppressor genes APC and TP53 and activating mutations in KRAS and phosphatidylinositol-4,5-bisphosphonate 3-kinase catalytic subunit alpha (PIK3CA) (38). The inactivation of the APC gene is believed to be one of the first mutational events that occur in the transformation of colon cancer (34). APC repression results in the Wnt signaling activation, followed by the nuclear translocation of β-catenin (39).

When Wnt is not present, cytosolic APC, axin, and glycogen synthase kinase 3 beta (GSK3β) interact with β-catenin. This phosphorylation module phosphorylates beta-catenin for degradation by the ubiquitin–proteasome pathway. Beta-catenin is unstable in the absence of Wnt signaling (40). Mutant APC indeed loses more than enough of its capacity for binding to this multiprotein destruction complex protein, allowing nuclear translocation of abnormal β-catenin, where it binds to a DNA-bound T-cell factor (TCF) and lymphoid enhancer factor (LEF) family of transcription factors (41). The Wnt/β-catenin target genes such as MYC, CCND1, VEGF, and PPARδ are implicated in tumorigenesis. These cells can mediate angiogenesis and proliferation (34). Mutations in other Wnt pathway components, such as AXIN1, AXIN2, or CTNNB1, in the absence of an APC mutation, may activate Wnt signaling and enhance tumorigenesis (34). The Wnt signaling pathway constitutes the principal intestinal epithelial cell proliferation modulator (42). Modulation of any element of this framework can alter the transcription of several other genetic materials and result in cancer.

The signaling pathway of Wnt is activated in virtually all CIN tumor types; APC mutations were identified in approximately 80% of them (43). The Colorectal Cancer Subtyping Consortium (CRC-SC) combined over several studies of gene expression from various preparation sampling methods and platforms into a comprehensive structure to improve the agreement between previous studies on colorectal tumor functional profiles (34). The Colorectal Cancer Subtyping Consortium (CRC-SC) classification, also known as the consensus molecular subtypes (CMS), categorizes four distinct categories (CMS1–CMS4) of colorectal tumors based on tested gene expression profiles. The CMS1 subtype is hypermutated and immunogenic. CMS2 tumors exhibit Wnt, MYC, and CIN activation, together with typical SCNA characteristics (34). CMS3 tumors are characterized by a metabolic cancer phenotype, while CMS4 tumors exhibit a strong stromal gene signature and are associated with the poorest survival outcomes (34).

### 3.2 Microsatellite instability pathway

Colorectal tumors can form through hypermutable pathways, which involve common somatic DNA base pair mutations, as opposed to the CIN pathway that involves frequent genomic copy number modifications (34). The microsatellite instability (MSI) pathway plays a key role in facilitating hypermutability. Changes in the DNA mismatch repair (MMR) genes (MLH1, MSH2, MSH6, and PMS2), or EPCAM and its regulatory protein, lead to instability in microsatellite sites (44). DNA microsatellites are formed of repeating sequences of mononucleotides and dinucleotides, as well as multimers that are repeated sequentially. DNA polymerase struggles to bind to repetitive genomic sequences, leading to mistakes in these regions (45).

### 3.3 Serrated pathway

Premalignant lesions for colorectal cancer are not solely derived from adenomas with internal tandem duplication (ITD). The diagnosis of serrated polyps can also be facilitated by advanced endoscopic technology, which also helps identify irregularly shaped lesions with diverse histopathologic appearances. Approximately 15% of colorectal cancer arising via serrated pathway are thought to originate from serrated polyps through serrated neoplasia (34).

Hyperplastic polyps (HPs) are the most common serrated polyps (composing approximately 67% of all lesions) and seldom develop into cancer (46). HPs have small bases with a brush border restricted to the top half and are characterized by predominant expression of predominant goblet cells, microvesicular and mucin-poor serrated polyps (MPSPs) (34).

Sessile serrated adenomas and polyps are the second most common type of serrated lesions, accounting for a quarter of all serrated lesions, while correct general incidence numbers are difficult to determine (47). A flat form, often overlooked under light visualization without chromoendoscopy, combined with subtle histopathological differences that can confuse interobserver consistency, even among skilled gastrointestinal pathologists, may complicate their identification (34).

The serrated pathway is an alternate mechanism for colorectal cancer. Serrated tumors with MSI exhibit rapid development from precursor to malignancy, similar to overall MSI-elevated cancers. Nguyen et al. (34) discovered that the serrated route is dependent on mutant BRAF with the inactivating mutation V600E in the mitogen-activated protein kinase pathway. BRAF mutations in microvesicular hyperplastic polyps indicate early activation of the serrated pathway. Mutation of one of these kinases may cause constipation and uncontrolled cell growth in the colon (34). There is a higher prevalence of BRAF mutations in sessile serrated adenomas than in conventional adenomas, suggesting that the serrated pathway is a colorectal cancer alternative (48).

### 3.4 CpG island methylator phenotype

Serrated neoplasia pathway tumors on either side of the colon may exhibit higher methylation at CpG islands. CpG islands are regions with high CG content, linked together by a phosphodiester bond. Gallardo-Gómez et al. (49) found enrichment across gene regulatory regions. Hypermethylation of promoter CpG islands at the 5' end of tumor suppressor genes can inhibit transcription, silence the gene, and lead to carcinogenesis. The molecular mechanism for hypermethylation remains unknown. MAFG, a transcriptional repressor, recruits BACH1, CHD8, and DNMT3B to cause hypermethylation of particular gene promoters, including MLH1 (34). Mutated BRAF upregulates MAFG protein and enhances promoter binding.

The CpG island methylator phenotype (CIMP) can be detected in early stages of carcinogenesis. Nguyen et al. (34) found that CIMP-high microvesicular hyperplastic polyps are more common than sessile serrated adenomas, traditional serrated adenomas (TSAs), and advanced lesions. A research study indicates that the status of CIMP is linked to ductal type, mucinous, BRAF mutations, and MSI in tumors, particularly in the right colorectal region in aged women (50). CIMP-positive cancers are associated with a poorer prognosis, particularly when they arise through the serrated pathway. CIMP is a reliable predictive factor, but it is not present in all cases.

MSI tumors can be triggered by methylation in the CpG-rich promoter region and become MMR-deficient, such as the MLH1 gene (51). MSI tumors with hypermethylation account for 75% of hypermutated colorectal cancer, while somatic mutations in MMR genes account for 25%. The CIMP and MSI have comparable approaches. CIMP-positive cancers have a high proportion of BRAF mutations and are often associated with serrated precursor lesions (35).

## 4 Role in redox homeostasis and cancer prevention

DNAs are influenced by various internal and external factors, leading to changes in physical or chemical composition (52). Although oxidative stress can damage cellular elements, the early occurrence of DNA damage and genomic instability may result in an upsurge of carcinogenic changes that promote cancer progression. Research has shown that the intrinsic mutagenic influence of reactive oxygen species (ROS) in DNA enhances the mutation frequency, thus facilitating carcinogenic changes in cells (53). DNA damage can be caused by ROS through various mechanisms such as base alteration, nucleotide depletion, loss of DNA structure, and crosslinking of DNA with proteins (54). Moreover, ROS, by triggering lipid peroxidation, can indirectly increase damage to cyclic DNA (55). The impairment of mitochondrial DNA bases is caused by oxidative stress and is crucial for mutations in mitochondrial genes (56).

The response of cellular DNA damage is a complicated process that includes various signaling pathways and proteins that are modulated differently across specific cancer types (57). After being exposed to different DNA-damaging agents, ranging from base alkylation and tumor cells to single-strand breaks (SSBs), the relevant DDR is activated to counteract these effects for their survival (58). According to research, ROS inhibited the activation of the DNA repair enzyme OGG1 (an 8-oxoG DNA glycosylase) by oxidizing critical cysteine residues such as Cys253 and Cys255 (59). Furthermore, ROS can delay the detection of damaged areas by affecting sensor kinases (ATM and ATR) and their downstream effector kinases (CHK1 and CHK2) (60). Apoptosis, a process conserved through evolution, serves for development and maintaining homeostasis. In addition, apoptosis is controlled through two primary outcomes, namely the death receptor and mitochondrial pathways, with ROS playing a role in the signaling of these pathways (61). Apoptosis is defined as high levels of ROS leading to oxidative stress and triggering the death of a programmed cell (62). This activation can occur through either the intrinsic (mitochondrial) or the extrinsic (death receptor) pathways. Minimal levels of ROS through the activation of various essential factors in the cell cycle also promote the growth of cancer cell lines. In tumors, the cells fail to react to normal apoptotic signals, resulting in uncontrolled growth (63). VB1 boosts ROS generation in anti-BRAFi melanoma cells, resulting in DNA damage, G2/M cell cycle halt, and cell death (53). Bcl-2 family proteins play a crucial role in the regulation of apoptosis, with their expression and mitochondrial relocation being influenced by ROS (64). ROS accumulation can activate the JNK pathway, which, in turn, stabilizes and activates p53, thereby promoting apoptosis (53). The cellular FLICE-inhibitory protein (c-FLIP) is a cytosolic protein that inhibits apoptosis triggered by death receptors. It was found that elevated levels of c-FLIP in multiple cancers can lead to increased resistance of tumor cells to apoptosis. ROS might influence protein conformation and promote protein degradation (65). Thus, inducing elevated levels of tumor ROS through chemical agents is believed to promote tumor cell apoptosis, thereby reducing tumor resistance to treatment.

Another type of cell death is programmed cell necrosis, which is regulated by dying receptors and carefully managed by signaling molecules of the intracellular. The mechanism entails programmed cell death by prompting the inhibition of apoptosis (66). Necroptosis is initiated through the activation of an upstream protein by receptor-interacting protein-3 (RIP3) that contains RHIM. This phosphorylation triggers mixed lineage kinase domain-like pseudokinase (MLKL), leading to harm to the necrosis and plasma membrane (67). Necroptosis can be triggered by ligands such as FasL and tumor necrosis factor (TNF), leading to swelling of the cell and bursting. Subsequently, it releases DAMPs, recognized as danger signals by the natural immunity, thereby initiating inflammation (68).

Autophagy is a process of intracellular degradation. Under conditions of stress or nutrient scarcity, the cell degrades damaged organelles and misfolded proteins, thus supplying the cell with energy (69). The role of autophagy, especially in tumors, is dual-natured and complex. Typically, tumors in the early phases of tumor progression are mainly inhibited by autophagy; at the same time, it promotes and sustains the survival of cancer cells (70). Research has shown that redox modification and signaling are closely linked to autophagy. For instance, free radicals will affect the activity of mTOR via several pathways, such as HMGB1, LKB1/AMPK, and PI3K/Akt, thus enhancing autophagy (71).

**Figure 1:**
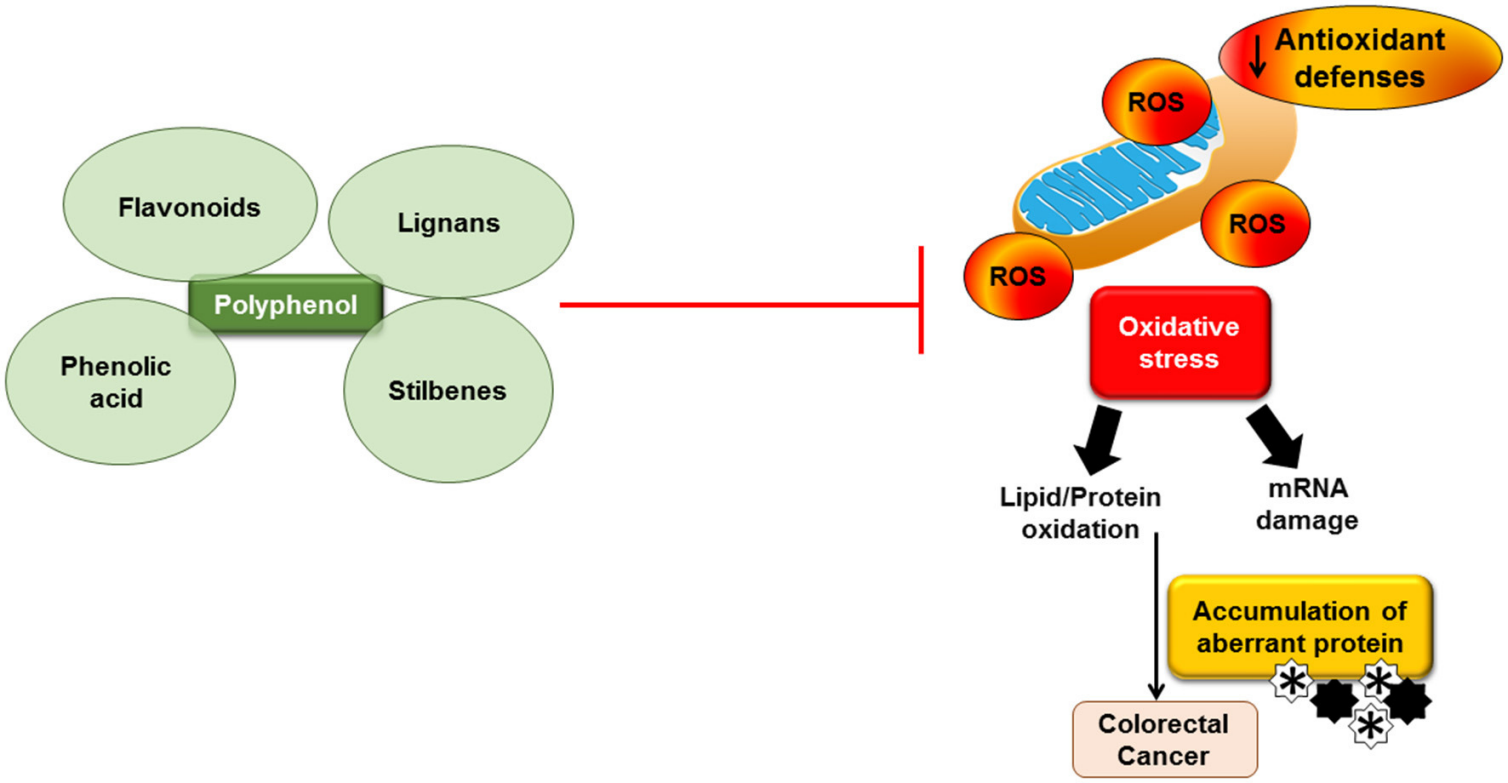
Effect of oxidative stress and the interaction of polyphenols in relation to colorectal cancer. Accumulation of reactive oxygen species (ROS) leads to oxidative stress and inflammation and subsequently causes mRNA damage and lipid/protein oxidation. Accumulation of aberrant proteins may contribute to colorectal cancer. Dietary polyphenols may block the ROS production.

In addition to the two characteristics of oxidative stress, which are involved in autophagy, it can also act in the opposite direction to mitigate oxidative stress through a negative feedback mechanism. ROS activation increases Ca^2+^ release through MCOLN1 stimulation and the nuclear movement of TFEB, which, in turn, boosts autophagy. Increased autophagy averts the buildup of surplus ROS (71). Failure to swiftly eliminate ROS through autophagy may indicate the demise of the cancer cell. In addition, there may be novel targets for addressing drug-resistant tumors, and more research is needed to identify the effect of oxidative stress on autophagy (71). Figure 1 shows the effect of oxidative stress and the interaction of polyphenols in relation to colorectal cancer.

## 5 Polyphenols

Polyphenols are natural substances of bioactive molecules derived from plants that have potent antioxidant effects, which have garnered increased attention in recent decades (4). Polyphenols are soluble in water, with a molecular weight between 500 and 4,000 Da. Polyphenols are the most common and abundant secondary metabolites produced by plants. Polyphenols are generally involved in protection against ultraviolet-induced damage and aggression by pathogens. In general, polyphenols are produced in nature via two primary pathways, which can occur autonomously or concurrently. One of these pathways involves the linking of two-carbon units, while the other one is the shikimic acid pathway. The characteristic of polyphenols is that they have at least one benzene ring with hydroxyl functions. Polyphenols can be either simple compounds or complex polymers (72).

Polyphenols can be classified into various classes according to the number of phenol rings, the chemical structure, the location of functional groups, or the carbon skeleton. Polyphenols mostly exist in the form of glycosides. The nutritional qualities and sensory qualities of plant foods, such as astringency, color, and odor, are partially influenced by the content of polyphenolic compounds in the polyphenols (73). In general, polyphenols can be categorized into four groups, namely phenolic acids, flavonoids, stilbenes, and lignans (74).

### 5.1 Flavonoids

Flavonoids comprise 2 aromatic rings and 15 carbon atoms linked by a 3-carbon bridge, including flavanols, anthocyanins, (iso)flavones, flavonols such as quercetin, flavanones, and chalcones (75). Flavonoids' fundamental structure consists of two benzene rings connected by a three-carbon chain containing one oxygen atom. They also typically exist as a form of aglycone or a form of flavonoid glycoside. This diversity in flavonoid molecular structure is due to the variations in the oxidation state and the hydroxylation pattern of the central pyran ring (75).

### 5.2 Phenolic acid

Phenolic acids are compounds that are made up of one phenolic ring and one organic carboxylic acid function (C6–C1 skeleton). Phenolic acids are divided into two categories, namely hydroxybenzoic acids, which encompass compounds such as gallic acid, and hydroxycinnamic acids, such as cinnamic, p-coumaric, ferulic, caffeic, and chlorogenic acids (75). Hydroxycinnamic acids usually occur as simple esters with hydroxy carboxylic acids or saccharides, while hydroxybenzoic acids primarily occur in glycosylated forms. Phenolic acids are chemically defined as hydroxylated derivatives of benzoic, cinnamic, phenylacetic, and phenylpropanoic acids (75).

### 5.3 Stilbenes

Stilbene is an organic compound, and its chemical structure is C6-C2-C6, which consists of two benzene rings (C6) connected by a two-carbon chain and usually has two isomeric forms (76). Resveratrol, piceatannol, and pterostilbene are groups of compounds from the stilbene family (76). Stilbene plays an essential function in photophysical, biomedical, and photochemical activity. Plants synthesize stilbenes, which are phytoalexins to protect themselves from bacterial and fungal growth (77).

### 5.4 Lignans

Lignans are a subgroup of plant polyphenols mainly found in seeds, cereals, whole grains, legumes, and fruits and vegetables. They are usually concentrated in the outer layer of grains, for example, the seed coat and pericarp. Lignans are diphenolic compounds made up of two phenylpropanoid units (C6C3) with β-β or C8-C8 linkages. The intestinal microflora hydrolyzes and metabolizes the lignans ingested by humans and animals, primarily resulting in enterolignans, as well as the compounds enterodiol and enterolactone (78).

## 6 Bioavailability and metabolism

The overall benefits of polyphenols in preventing cancer, especially colorectal cancer, cannot be understood as solely conditioned by the level of their own biochemical activity. Bioavailability in terms of the ratio of absorption and transfer to usable tissue in an active state is just as conclusive. While *in vitro* studies are constantly confirming strong anticancer effects by dietary polyphenols, this promise is hampered by multifaceted aspects of absorption, metabolism, and transport (79).

After intake, dietary polyphenols are largely found in conjugated forms, for instance, glucosides, esters, and other variants, which are poorly absorbed during the passage through the duodenum and proximal jejunum. A small amount of these compounds also diffuses through enterocytes in passive ways or is taken into portal circulation by the specified membrane transporter systems. The majority of the consumed polyphenols arrive in the colon, where they are derived through microbial degradation into smaller phenolic acids together with a range of other products (80). These colonic metabolites that most often display bioactivities equal to or more potent than the parent compounds have a dominant effect to inhibit the proliferation of the intestinal cells in the mucosa. In experimental rodent models, for instance, green tea catechins and hazelnut polyphenols are bioconverted to gallic acid, catechin conjugates, and urolithins, which are more resistant to degradation and are retained more efficiently in colonic tissues (81).

After oral consumption, polyphenols are metabolized significantly in the liver through phase II transformations, that is, the glucuronidation process, sulfation, and methylation. These responses reduce the anticancer and antioxidant activity of these compounds in the body circulation (82). This could be seen in curcumin, a compound with great pharmacological potency but low oral bioavailability, which by far experiences a fast conjugation of hepatic processes, bringing its bioaccessibility down drastically (83). Smart conjugation of resveratrol and quercetin with glucuronic acid derivatives results in the rapid formation of sulfates and glucuronides, respectively, thereby maintaining their concentrations at low active levels. Therefore, they can only be effective when measures are taken to increase their uptake and memorization (84).

Colorectal cancer constitutes a major health challenge, with the available therapies characterized by low efficacy; thus, studies have been aimed at enhancing the bioavailability of polyphenolic chemopreventive agents. Various strategies such as the new development in formulation technology and modalities of delivery have been tested through experimental models. One strategy that has been extensively explored is the use of nanoparticles, for instance, liposomal curcumin or nanocarriers filled with resveratrol, which protect polyphenols against premature metabolism and promote their intestinal absorption (85). These nano-formulations result in significantly higher levels of active compounds reaching colonic tissue, leading to improved tumor prevention in murine models of colorectal cancer (86). Co-administration with bioenhancers has been effective in absorption. Piperine also plays a crucial role by blocking hepatic metabolism; thereby, this bioenhancer increases the plasma levels of curcumin (87). In addition, dietary fat positively influences the uptake of lipophilic polyphenols such as carotenoids and quercetin (88).

## 7 Role of polyphenol-rich foods in the prevention of colorectal cancer

### 7.1 Green tea catechins

Polyphenols are a diverse group of naturally occurring compounds found in fruits, vegetables, teas, and spices, and they have received a lot of attention for their potential chemopreventive and therapeutic effects in the field of colorectal cancer. Several preclinical investigations have been conducted *in vitro* (cancer cell lines) and *in vivo* (animal models) to assess the efficacy and molecular basis. Recent scientific studies have shed light on the possible function of green tea, particularly its major catechin, epigallocatechin-3-gallate (EGCG), as a preventative and therapeutic ingredient in colorectal cancer. The mechanisms of EGCG anti-cancer activity are based on the properties of apoptosis induction, cell proliferation arrest, angiogenesis inhibition, and influence on inflammatory and oxidative pathways, which are mediated by the nuclear factor-kappa B (NF-κB), STAT3, and PI3K/AKT/mTOR axis (89). In another set of *in vitro* investigations using various colorectal cancer cell lines (SW480, HCT116, and HT29), it was shown that EGCG inhibited cell growth, elevated apoptosis-related caspase, and downregulated anti-apoptotic proteins such as Bcl-2 and MCL-1 (90).

The chemopreventive effect of green tea components is supported by *in vivo* investigations using animal models. The results of the research showed that green tea polyphenols caused dose-dependent tumor growth regression, decreased expression of vascular endothelial growth factor (VEGF), and reduced activity of cancer stem cells (91). Epidemiological research also indicates that the primary polyphenol, EGCG, primarily triggers antioxidant and pro-oxidant actions, which selectively enhance oxidative stress levels in neoplastic cells, driving apoptotic consequences (92). Green tea has potential as a dietary agent against colorectal cancer due to the multifactorial pathways of the activity that drive its effect. Moreover, it is non-toxic and has a pathway-wide therapeutic potentialization, which enhances its appropriate applicability in oncological medicine as a complementary therapeutic agent.

Research conducted in China and Japan, where green tea is widely eaten, has consistently shown a decrease in colorectal cancer occurrence with continuing consumption of green tea. South Korean, Indian, and Taiwanese research investigations offered more *in vitro* and human data on green tea's antineoplastic properties (91). European cohorts, specifically Italy and the United Kingdom, have participated in meta-analysis studies to examine the relationship between dietary polyphenols and colorectal cancer risk, while the United States has conducted clinical trials evaluating green tea extract in high-risk individuals (91). When combined with the other international efforts, the application of green tea in preventing colorectal cancer internationally has been demonstrated, and additional randomized controlled trials are needed to determine the best dosage, length, and population impact.

### 7.2 Curcumin

Curcumin is a bioactive monomer derived from *Curcuma longa* (93). The comprehensive body of research conducted *in vitro* demonstrated that curcumin induces anti-inflammatory, antioxidative, and anti-proliferative effects, thus becoming an effective phytochemical agent (94). Furthermore, it inhibits migration and invasion by adjusting the Wnt/β-catenin and MAPK signaling pathways (95). Such effects are mediated, in part, through the curcumin-mediated downregulating effect on cyclin and CDK and concurrent upregulating effect on CDKI, both of which are associated with its growth-inhibitory effect on a wide range of cancer cells (96). Quercetin, a flavonoid found in onions and apples, inhibits cell proliferation, reduces expression of metastasis-related proteins MMP-2 and MMP-9, and weakens the oxidative stress signaling network COX-2 (97).

In addition to curcumin, the combination of curcumin therapy with irinotecan (IRI) (2.5–20 μM curcumin + 10–100 μM IRI) has been shown to diminish chemoresistance in the LoVo and irinotecan-resistant LoVo/CPT-11R cell lines under preclinical circumstances (98). An increase in effectiveness was linked to the suppression of cancer stem cell markers by CD44 and EpCAM, as well as the concomitant decrease in pro-survival proteins by Bcl-2 and activation of the pro-apoptotic protein Bax (99). The epithelial–mesenchymal transition (EMT) process and its regulation, which is a critical process during cancer metastasis, have been reported to be regulated by curcumin through increasing the expression of E-cadherin and decreasing the expression of vimentin and N-cadherin at the same time (100). With colon cancer, it exerts combined synergistic effects with the first-line chemotherapeutic cytostatic agent oxaliplatin (OXA), causing both resistant and sensitive cell line proliferation inhibition and apoptosis. The synergy of this combination is especially striking in the line becoming resistant to OXA, the effect that is attained via curcumin-induced inhibition of the TGF-/Smad2/3 signaling cascade (98).

Another area of research leads to the combination of regorafenib (RG), which is a multikinase inhibitor, with curcumin. This mixture has a strong antitumor activity in colon cancer xenografts; especially, it is capable of inducing perfect tumor regression at a low dose (101). In HCT116 cells, the combination of the KRAS mutation greatly boosted apoptosis and autophagy activity. Curcumin appeared to have MEK-inhibitory activity, implying a function as a MEK-inhibitor complement to traditional treatment (0–8 μM curcumin + 0.5–32 μM oxaliplatin) of KRAS-driven malignancies that are intrinsically resistant to these drugs (98).

The process of carcinogenesis in colorectal cancer is controlled by numerous pathways, one of which is the Wnt cascade, already proven to be essential (93). The majority of CRC patients have mutant alleles of genes coding Wnt signaling components, especially APC and β-catenin. In Ojo et al.'s study (93), a CRISP-based functional screen found that the cell line SW480 was shown to have attenuated proliferation under curcumin treatment, which is associated with the reduced expression of miRNA-130a and the Wnt/β-catenin pathway. These findings suggest that the regulation by miRNAs is an important factor that defines the antitumor effect of curcumin. This conclusion was further corroborated by the fact that CRC cells were partially rescued by the exogenous expression of miRNA-130a against curcumin-induced growth arrest.

Extracellular degradation enzymic regulators partly mediate CRC invasion, such as an enzyme-type plasminogen activator (uPA) and matrix metalloproteinase-9 (MMP9) (96). These enzymes stimulate AMPK and have inhibitory effects on NF-κB, which acts on the cancer cells to sustain their spread. In addition, curcumin inhibited the expression of NF-kB to render drug-resistant CRC cells to oxaliplatin (OXA) *in vitro* (98). The dual effect of curcumin and OXA led to a strikingly higher degree of cell death and colony formation compared to each of the components.

CRC is a dominant clinical concern of metastatic development that attacks the liver. Weng and Goel (102) reported a decrease in the size of hepatic metastases after treatment of the CRC-derived liver lesions in the athymic nude mice with curcumin. Intriguingly, Ma et al. (103) demonstrated the safe pharmacokinetics of curcumin in a phase II clinical trial of the adjuvant role of this substance in combination therapy with chemotherapy. These data infer that curcumin is a potentially feasible and well-tolerated approach for patients with metastatic disease.

### 7.3 Resveratrol

Resveratrol is a stilbenoid found in grapes, peanuts, and mulberries that causes apoptotic cell death by depolarizing the mitochondrial membrane potential and producing reactive oxygen species (104). It can inhibit cancer by targeting numerous signaling pathways, including AMPK, ROS, NF-κB, and caspases (105).

Chronic inflammation is one of the key causes behind CRC. Anti-inflammatory agents may thus be useful in the treatment of CRC. Damaged tissues rapidly release cytokines, which initiate the inflammatory response (96). Previous research has shown that intestinal cells exposed to cytokines can activate inflammatory pathways such as the JAK-STAT, NF-kB, and MAPK cascades; additionally, pro-inflammatory enzymes, pro-inflammatory mediators, and reactive oxygen species (ROS) can be produced (96). Resveratrol reduces pro-inflammatory mediators (TNF-α and IL-1β), enzymes (iNOS and COX-2), and signaling pathways (NF-kB) (106). Pterostilbene (trans-3, 5-dimethoxy-4′-hydroxystilbene), which is structurally similar to resveratrol, inhibits the p38 MAPK signaling pathway, hence inducing COX-2 and iNOS. This anti-inflammatory impact can help prevent colon cancer (96). Resveratrol increases inflammation, decreases neutrophils in the lamina propria and mesenteric lymph nodes, and regulates CD3(+) T cells that produce TNF-α and IFN-γ. Furthermore, observations show that resveratrol reduces the inflammatory marker P53 (96).

### 7.4 Quercetin and other flavonoids

Sweet potatoes, onions, okra, kale, blueberries, peaches, blackberries, grapes, cherries, and plums contain quercetin, one of the flavonoid molecules (107). Quercetin inhibits colorectal cancer by altering numerous molecular pathways. Quercetin-induced apoptosis in colorectal cancer cells was associated with downregulation of the Wnt/β-catenin pathway and gene expression (cyclin D1 and survivin) (96). Quercetin may inhibit colorectal cancer cells by regulating NF-κB, JNK/JUN, and PI3K/AKT/mTOR (108). A study found that quercetin's anti-inflammatory properties helped to prevent colorectal cancer. This led to decreased tumor growth, reduced inflammation, and downregulation of oxidative stress markers (96).

Anthocyanins, found in berries and other colored vegetables, promote the production of pro-apoptotic proteins while suppressing the action of oncogenic signaling pathways such as PI3K/Akt and MAPK in both HCT116 and Caco-2 cell lines (98). In another study, Ko et al. (109) found that gallic acid and its derivatives inhibited cell cycle progression, activated apoptotic processes via ROS generation, and damaged DNA through contact with G-quadruplex DNA complexes.

Cellular homeostasis is maintained through a constant cyclical process governed by cyclins, cyclin-dependent kinases (CDKs), and cyclin-dependent kinase inhibitors. A recent study has added to the evidence that quercetin accelerates cell cycle arrest by regulating several target proteins, including p53, p21, p27, cyclin B, cyclin D, and cyclin-dependent kinase, all of which act together to control and regulate mitosis. Quercetin, in particular, promotes G2/M phase arrest by preferentially increasing p73 and p21, as well as cyclin B at the transcriptional and translational levels (96). According to Rather and Bhagat (110), quercetin has the ability to downregulate cyclin B1 and cyclin-dependent kinase-1 (CDK-1), hence interrupting G2/M progression. Furthermore, quercetin-induced cell arrest is occasionally recognized as an aggregation of p21 and retinoblastoma protein (pRb), suppressing transcription factor E2F1 activity at the G1-S boundary (96). Indeed, the exact mechanism of quercetin-mediated cell growth arrest varies by cell type and may occur during the G1 phase.

### 7.5 Piperlongumine

Piper longum is a long pepper fruit, and its amide alkaloid piperlongumine (PPL) was characterized in 2021 as 5,6-dihydro-1-[(2E)-1-oxo-3-(3,4,5-trimethoxyphenyl)-2-propenyl] (111). According to epidemiological research, PPL has chemopreventive effects against oral, breast, liver, kidney, stomach, pancreatic, and colon cancers; bladder lymphomas; and melanomas (112). These biological effects are linked to three mechanisms, for instance, activation of the suppressor of mothers against the decapentaplegic family member 4 (SMAD4) pathway, downregulation of antioxidant enzyme activity, resulting in an increase in ROS levels, and an antimetastatic effect (112). PPL stimulates ROS generation within tumor cells, and this oxidative overload causes cell death via the caspase- or Ras-controlled pathway (113). Overexpression of SMAD4 facilitates a separate pathway that boosts the production of the senescence-promoting p21 transcription factor while suppressing the anti-apoptotic proteins B-cell lymphoma 2 (BCL2) and survivin, inducing cellular senescence and death. Taken together, these findings demonstrate that the ROS/Ras/Akt and SMAD4 signaling pathways are linked to PPL's colon tumor-killing action (111). Table 1 summarizes the role of dietary polyphenols in preventing cancer.

**Table 1:** Studies conducted on dietary polyphenols and their effects on cancer.

**Polyphenols**	**Dietary source**	**Demonstrated effects**	**Mechanisms of action**	**Reference**
Epigallocatechin-3-gallate	Green tea	Inhibited colorectal cancer cell lines (SW480, HCT116, and HT29)	EGCG inhibited cell growth, elevated apoptosis-related caspase, and downregulated anti-apoptotic proteins such as Bcl-2 and MCL-1	(90)
		Induced dose-dependent tumor growth regression	Decreased expression of vascular endothelial growth factor (VEGF) and reduced activity of cancer stem cells	(91)
		Enhanced oxidative stress levels in neoplastic cells	Triggers antioxidant and pro-oxidant actions	(92)
Quercetin	Onions and apples	Inhibits cell proliferation	Reduces expression of metastasis-related proteins MMP-2 and MMP-9 and weakens the oxidative stress signaling network COX-2	(97)
Curcumin	Turmeric	Attenuated proliferation of SW480 cells	Reduced expression of miRNA-130a and the Wnt/beta-catenin pathway	(93)
Curcumin	Turmeric	Decrease in the hepatic metastases size after treatment of the CRC-derived liver lesions in the athymic nude mice	Suppress TNFα- or IL-1β-induced p38 and JNK activation, causing the eventual suppression of IκBα degradation in HT29 cells	(102)
Resveratrol	Grapes, peanuts, and mulberries	Inhibited colorectal cancer cell viability	Induces human colorectal cancer cell apoptosis by activating the mitochondrial pathway via increasing reactive oxygen species	(95)
Quercetin	Sweet potatoes, onions, okra, kale, blueberries, peaches, blackberries, grapes, cherries, and plums	Induced apoptosis in colorectal cancer cells	Downregulation of the Wnt/β-catenin pathway and gene expression (cyclin D1 and survivin)	(96)
Quercetin	Sweet potatoes, onions, okra, kale, blueberries, peaches, blackberries, grapes, cherries, and plums	Inhibit colorectal cancer cells	Regulating NF-κB, JNK/JUN, and PI3K/AKT/mTOR	(108)
Anthocyanins	Berries and other colored vegetables	Suppressed HCT116 and Caco-2 cell lines	Promote production of pro-apoptotic proteins while suppressing the action of oncogenic signaling pathways such as PI3K/Akt and MAPK	(98)
Gallic acid	Walnuts, almonds, red wine, and green and black tea	Hindered A549 lung cancer cell progression	Inhibited cell cycle progression, activated apoptotic processes via ROS generation, and damaged DNA through contact with G-quadruplex DNA complexes	(109)
Piperlongumine	Long pepper fruit	Chemopreventive effects against oral, breast, liver, kidney, stomach, pancreatic and colon cancers; bladder lymphomas; and melanoma	Activated suppressor of mothers against the decapentaplegic family member 4 (SMAD4) pathway and downregulated antioxidant enzyme activity	(112)

## 8 Clinical evidence and human trials

The European Prospective Investigation into Cancer and Nutrition (EPIC) cohort aligns with recent epidemiological studies; it is prospective, longitudinal, multinational, and includes over 521,000 participants across 10 European countries (114). A recent examination of this group of people revealed that individuals who had the highest consumption of flavonoids and lignans showed a significantly lower prevalence of colon cancer, which was especially notable in women and those who regularly ate according to a Mediterranean-style diet (115).

Population-based studies in Asia, the Middle East, and North America, such as epidemiological findings, strengthen the connection between the intake of polyphenols and a reduced incidence of colorectal cancer. Epidemiological studies conducted in Japan and South Korea, where green tea, soy isoflavones, and fermented vegetables are common, also showed that the intake of dietary polyphenols is linked with a reduced incidence of colorectal cancer (96). There is supportive prospective cohort evidence that the use of polyphenol-rich diets, especially those of traditional Iranian and Mediterranean diets, is inversely commensurate with a risk of colorectal cancer, reflecting the global importance of polyphenol-rich nutrition (116).

The randomized controlled trials involving 41 subjects have focused on individual polyphenol compounds or polyphenol-rich extracts (2 g or 4 g curcumin per day for 30 days) and have been investigated with regard to their ability to modulate procarcinogenic eicosanoids prostaglandin E_2_ (PGE_2_), 5-hydroxyeicosatetraenoic acid (5-HETE), and Ki-67 that have been shown to disparage the risk of colorectal cancer; this includes oxidative DNA damage, inflammation, cell growth, and recurring adenoma formation (117). As shown above, 6 months of daily curcumin supplementation (4 g) significantly decreased the number of aberrant crypt foci and adenomatous polyps in patients with a previous history of colorectal neoplasia to a significant extent. In a similar study conducted in 2024 by de Oliveira Assis et al. (118) on patients aged 18 to 66 years (15 randomized controlled trials) with familial adenomatous polyposis (FAP), it was found that green tea catechins EGCG ( ≤ 8 weeks) reduced serum levels of TNF-alpha and other oxidative stress indicators, and this compound, thus, exhibited anti-inflammatory and chemopreventive effects. Furthermore, these randomized controlled trials also emphasize the translational value of preclinical phenomena and define the feasible dietary lessons on colorectal cancer prevention or risk reduction.

Observations made in cohort studies involving observational research designs have yielded longitudinal results on dietary behavior and cancer in non-manipulative situations. The Southern Community Cohort Study prospectively enrolled individuals from the southeastern United States during 2002–2009, involving 71,599 participants, and has proven that participants with high intakes of total polyphenols, tyrosols, and hydroxybenzoic acids in their diets (587–1597 mg/day) showed considerably lower occurrences of colorectal cancer or rectal cancer (119). Similar findings can be observed in the European cohort, EPIC, and the Korean cohort, KoGES, which concludes that following a diet rich in polyphenols is associated with a decreased incidence of colorectal cancer even when traditional confounders (age, body mass index, and red meat consumption) are considered (120).

## 9 Challenges in translating findings to humans

Although numerous *in vitro*, animal, and epidemiological studies indicate the anticancer effects of dietary polyphenols, it remains difficult to extrapolate relevant and evidence-based methods for colorectal cancer prevention in people (121). Due to biological, methodological, and practical challenges, current information is not fully applicable in clinical practice.

The main difficulty is that the majority of polyphenols have minimal oral bioavailability. Curcumin, quercetin, and resveratrol are antioxidants with high *in vitro* activity that are degraded, conjugated (for instance, glucuronidated and sulfated), and excreted, resulting in low systemic quantities (83). Furthermore, the majority of polyphenols enter the colon in metabolized form or are transformed by intestinal bacteria into secondary metabolites, the biological activity of which can differ greatly from that of the parent substances (119). Because of the drug's metabolic diversity, it is challenging to determine a suitable dose, formulation, and frequency of administration for therapeutic benefits in the human gut (122).

Individual differences in polyphenol metabolism provide a significant difficulty to providing precise consumption recommendations. Genetic polymorphisms of enzymes that transfer metabolism, various gut microbiota compositions, various dietary patterns, and associated morbidities have an impact on absorption and metabolism (123). As a result, even when an individual consumes the same amount of food, the plasma or colonic concentration of a certain polyphenol, for example, epigallocatechin gallate, can vary significantly. Such biological diversity makes it harder to set standardized standards and limits scientists' capacity to forecast who will benefit the most from polyphenol-based therapies (124).

Furthermore, clinical trials with polyphenols have substantial methodological problems. Many trials use various polyphenol types, sources (extracts vs. whole foods), administration techniques, and biomarker outcomes, resulting in variable results (125). Some studies use surrogate endpoints such as oxidative stress markers or polyp counts rather than long-term cancer incidence or survival, which limits their therapeutic usefulness (126). High-dose supplementation, such as β-carotene in smokers, has been linked to increased cancer risk rather than reduced risk (127), raising questions about compliance and long-term safety. There are several limitations that hinder the interpretation of clinical trial findings, including small sample size, variability in polyphenol dosage, short treatment duration, potential confounding factors such as age, and possible bias from the interpreting pathologist. Moreover, the dose–response relationship in cancer has not been adequately evaluated.

## 10 Conclusion and future perspective

Oxidative stress, resulting from excessive production of ROS due to an imbalance between oxidants and antioxidants, has been implicated in the upregulation of oncogenes and the formation of mutagenic compounds, thereby promoting proatherogenic processes and inflammation. Dietary intake of foods rich in polyphenols plays an essential role in supporting immune function, sustaining cellular energy production, and scavenging ROS. The wide range of biological processes mediated by polyphenols suggests their protective role in the pathogenesis of colorectal cancer. Current preclinical studies support polyphenols' intrinsic anti-colon cancer activity and demonstrate their complicated mode of action, which includes impacts on signaling pathways, transcriptional control, and cellular oxidative stress. These findings establish a scientific truth, and the study of polyphenols will supplement traditional chemotherapy drugs to reduce resistance and toxicity associated with chemotherapy treatment. However, the poor water solubility and low physicochemical stability of polyphenols have limited their intestinal absorption and overall bioavailability. Nanotechnology has emerged as a promising platform for drug delivery. It has gained significant attention for its potential to overcome limitations associated with conventional therapeutic agents, including poor water solubility, lack of target specificity, systemic toxicity, and non-specific biodistribution. The application of nanotechnology can enhance drug efficacy and bioavailability by improving solubility, prolonging plasma half-life, protecting active compounds from degradation in the gastrointestinal environment, and increasing intestinal permeability. Current data on the interactions between chelating agents and polyphenols remain limited. Further investigation may facilitate the development of potent therapeutic agents and novel biomarkers that target cancer tissues and elucidate the downstream mediators involved in oxidative stress pathways. Although polyphenols may not function as pharmacological agents, they hold considerable promise and may provide leads for future strategies to combat colorectal cancer. Their potential as adjuncts or alternatives to conventional therapies warrants further investigation through long-term clinical trials.
